# Correlation between lipid profiles and body mass index of adolescents obesity in Padang

**DOI:** 10.1186/1687-9856-2013-S1-P87

**Published:** 2013-10-03

**Authors:** ES Zul Febrianti, Asviandri, Lita Farlina, Rahmi Lestari, Susetyo Cahyohadi, Eka Agustia Rini

**Affiliations:** 1 Endocrinology Division, Pediatric Health Departement of MedicalFacultyAndalasUniversity, Dr.M.DjamilHospital Padang

**Keywords:** obesity, lipid profile, body mass index

## Background

Obesity is an emerging public health problems throughout the world where it is on the increase during childhood, adolescence and adulthood. Obesity is associated with hypertension and dyslipidemia caused by changing eating pattern, life style and activities. Obesity could give negative effect of cardiovasculair disease and metabolic syndrome.

## Objective

To determine correlation between blood lipid profile and body mass index of adolescents obesity in Padang.

## Methods

The study was part of the study of obesity for adolescents in Padang 2010 with cross sectional study of anthropometric measurement (body weight, body height and body mass index), versus blood lipid (cholesterol, LDL, HDL, Triglyceride) serum. The data were analyzed by SPSS 19 using Chi square, T test independent and regression correlation of Pearson’s method.

## Result

The samples included 36 male (45%) and 44 female (55%) adolescents obesity with mean of ages 16 years 4 months. Mean of body mass index was 32,02(SD 3,47). The dyslipidemia; hypercholesterolemia, increased LDL, increased of triglyceride and decreased of HDL (40%, 41,3%, 25% and 43,8% respectively). The triglyceride level increased with increasing BMI (p = 0,008 and R = 0,294), but there were no significant correlation between cholesterol, HDL and LDL versus body mass index (p = 0,258; 0,416 and 0,594; with R = 0,128; 0,09 and 0,06 respectively).

## Conclusion

There was a positive correlation between body mass index and triglyceride level.

